# Observations on General Paresis

**Published:** 1896-01

**Authors:** F. H. Stephenson

**Affiliations:** Syracuse, N. Y.


### OBSERVATIONS ON GENERAL PARESIS.

By F. H. STEPHENSON, M. D., Syracuse, N. Y.

I WILL invite your attention for a few moments to the consideration of general paresis of the insane, with reports of several cases which have fallen under my observation, presenting many phases, some having been seen in their incipiency, when but little physical or mental change was recognisable.

The question is often asked, Does general paresis exist without mental disorder? I have seen several cases which continued for months without exhibiting mental deterioration and I have found reports of many others; but I know it is the accepted opinion that mental changes are found in most instances, before

---

1. Read at the twenty-eighth annual meeting of the Medical Association of Central New York, October 15, 1895.

death, though often presenting symptoms only of weak-mindedness and little importance is attached to them.

The rapidity of degeneration and the amount of mental loss in a given time, of course, varies. According to Savage, the disease is special in so far that it ends fatally in nearly all cases and whatever the earlier symptoms may have been; those of the later stages are similar to a remarkable degree. Remissions do occur and patients improve to so great an extent that they are discharged from hospitals, and if they lead a quiet life they remain well for months, but usually not over a year. If they engage in active pursuits the breakdown comes sooner and a second return to health rarely occurs. The greatest importance attaches to the early stages, for if anything is to be accomplished in treatment it is at this time. A paretic will make unreasonable investments bringing on financial ruin and his condition not be recognised until his financial fall. Friends say his losses caused him to lose mental balance, when, in reality, his mental failure preceded and was the cause of his wrecked finances. On this account early attention and proper restrictions should be made.

The prodromal symptoms are of vast importance, enabling us to make an early diagnosis. They may be physical or mental. Headaches and attacks of vomiting often precede this disease. When head-pains of a variable kind occur with men usually strong, unless of strongly neurotic families, it is well to be guarded in the diagnosis. If with such men there is a cessation of business and family cares, the onset of paresis can often be deferred. Word blindness and word deafness or over-acute hearing are sometimes met with; clipping of words or letters; difficulty in pronunciation; slurring them over indistinctly; tremor of the tongue and facial muscles, especially about the mouth; over-action and twitching of the occipito-frontalis—these are all suspicious symptoms.

Dr. Gray suggests extending the arm and leg, then letting them rest on your hand, will readily reveal a tremor, which, in many cases, has not been observed. The wrist and knee reflexes are exaggerated, though rarely are they diminished. I have observed that the pupillary alterations are frequent and vary greatly, some being irregular in their marginal contour, some dilated. Rarely are they contracted; often one is larger than the other; there may be sluggish reaction to light and often they do not react to accommodation. Ataxic gait develops, also ataxic movement of the upper extremities, which is well shown by having the patient close his eyes, extend and swing his arm, then suddenly tell him to touch the end of his nose with the index finger. I have repeatedly seen this incoördination thus brought out.

With the physical symptoms we earlier or later observe mental changes, as great irritability, forgetfulness, marked extravagance, irregularities in business matters and suspicion, until the patient develops the second or maniacal stage. In this stage the mental symptoms increase ; there are stupor and delusions of a grandiose character, the patient imagining himself very rich and powerful, but being too dull to reason his point. This form differs from the mania of paranoia where the delusions are sustained by argument. In this stage they often become violent and die of exhaustion, or commit suicide, especially if at all melancholic.

In the third stage or stage of dementia they become foolish and silly, showing lack of all mental process.

*Duration.*—These cases average, according to many reports which I have gathered, about two years. This applies more strictly to those of whom a specific history was given, though some cases live from four to six years after the earliest noticed symptoms. Convulsions may occur during the disease. In some of my cases there was but slight mental change noticed until after the convulsions appeared ; in fact, in one case it appeared to be the onset and there was no recurrence of convulsions until within a few hours of death, which occurred nearly a year after the first attack. With this man the symptoms were largely physical—there was mental enfeeblement, but no delusions or ideas of grandeur.

Any condition which may start a decay of the higher nervous tissue will give rise to symptoms which resemble those of paresis. Moreover, it may be a matter of accident whether this be due in the first place to disease of arteries, to malnutrition with constant strain or changes in relation between brain and blood-vessels.

Regarding the relationship between syphilis and general paralysis, I have found several cases reported where that disease was followed in both husband and wife by paresis. I have had under observation for some time two women who suffered from Jacksonian epilepsy, both having had syphilis. The spasms yielded entirely to specific treatment ; their husbands had both had syphilis and have recently died of general paralysis.

The question regarding etiology often arises, but what is general paresis ? Certainly it is a premature irregular dissolution, differing from senility in its irregular manifestations both mental and physical, the changes in some cases being rapid, in others explosive, while some are very slow and present marked remissions. An imperfect brain, either hereditary or from congenital causes or imperfect development, it appears to me, must be the first condition in resultant degeneration in a large number of paretics. Although this statement is probably doubted by many observers, I cannot see why the many causes given—namely, syphilis, intemperance, overwork, worries, bodily diseases or infirmities, traumatism and exposure, may not be the excitant ones, as I believe they are in many cases, though not the primary causes in all.

We often meet with paretics in whom all of these familiarly so-called causes are absent and their histories differ greatly, some presenting mere physical changes with little mental disturbance, others in whom the ego is wonderfully altered and exalted with little physical deterioration. Some asylum statistics, I am aware, go to prove Morel's early statement that heredity or a strong neuropathic constitution does not enter largely into the etiology of paresis. Regarding the temperature changes, I think most carefully prepared statistics have proven that the principal variations are due to different forms of paresis, but more especially to the development of some intercurrent affection as inflammation of the respiratory tract, bed sores or the onset of convulsive seizures.

One very interesting and I might say typical paretic whom I observed for three years, during the entire duration of his disease presented a most remarkable pulse. At intervals during the first three months of illness the beats dropped to thirty per minute and so continued for two or three hours each time. Severe epistaxis was also frequent in this case, though the heart failures were not associated with the hemorrhages, occurring frequently on different days. It was probably due to some derangement of the cranial branches which help to form the cardiac plexus, as there was no apparent valvular disease, though such is often found where syphilis is associated if not the cause, as was the condition in this patient. I have had some very interesting cases of general paresis under treatment, combining also tabetic symptoms in the legs. In one the arms, head and tongue presented the jerky incoördination of disseminated sclerosis. As yet there has been no opportunity of demonstrating the two lesions.

The history and symptoms are as follows:

Mrs. W., aged 45; married; no children. Family history negative, so far as I could learn. Previous health always good until one year ago, when she had la grippe; since then never well. The first and most attractive sign was the marked tremor of the head and exaggerated or overaction of the facial muscles when she attempted to speak. There was almost a snapping of the jaws. Loss of memory; very nervous and restless, but fairly intelligent. Says she sees shadows following her and hears voices talking to her, which two latter symptoms are very suggestive of insanity. There was no swaying of the body with the eyes closed. She could walk, also walk well, when the eyes were closed. Good grip; ataxia of the upper and lower extremities; knee-jerks absent. Her family say she was formerly always good-natured, but has become fretful and is often very irritable. She is very weak and has headaches very often in the morning, but during the day they disappear and she feels stronger. Sleeps well; no vomiting; good appetite; at times very stupid and stumbles when walking. In this case the flushings of the face and pallor, vasomotor phenomena, are very marked.

Of fourteen *postmortems* held during the past year and reported by Dr. F. C. Sawyer, of the St. Lawrence State hospital, the calvaria were thickened in seven cases; thin in three cases; firmly adherent in three cases; the condition in the fourteenth not mentioned. The dura was thickened in seven cases; firmly adherent in three cases; the condition not reported in the remaining four. The pia was thickened in five cases; increased and adherent vessels in four cases; opaque in one; attenuated and anemic in one case. The arachnoid was thickened, opaque, infiltrated and distended with fluids in about half the cases. The cortex was quite generally softened, with but little atrophy and irregularly located erosions on all the convolutions of the vertex; the basial ganglia bulb and olfactories were softened. Also an overgrowth of the neuroglia, and atrophy of the cells and nerve fibers of the gray matter; ventricles enlarged; vascularity increased. The lumen of the vessels is diminished, the muscular coat hypertrophied and partially paralysed and degenerated, allowing extravasation of blood into surrounding tissues. Development of spider cells, which proliferate and develop, according to Lewis, into scavenger cells. The weight of the brains was given, the variation being from thirty-seven to fifty-three and one-half ounces. In the spinal cord changes were found similar to those in the brain. The posterior commisure, the columns of Goll and Burdach, are most often affected and present the changes of descending degeneration. Anesthesia of the skin is occasionally present and, as Dr. Burns stated, leads to self-mutilation. Patients think the limb or portions of the body affected is dead or a foreign body. One of my patients wished his limb taken off, insisting it was a fish leg.

A few cases of Charcot artropathies have been reported in general paretics. I saw one in the National hospital of London, associated with paresis and tabes dorsalis, and recently one in the St. Lawrence State hospital in Ogdensburg. In the latter case ptosis of the left eyelid also was present with the Charcot lesion of the left knee. It was a transferred case, the early history of which was not at all satisfactory, and as the patient was in the last stages of paresis, his information regarding development was valueless. Optic atrophy I have also observed in three cases causing blindness ; this I think is rare before the latter stages. The pathological changes in the thoracic and abdominal cavities vary greatly, though Julius Mickel reports pulmonary disease in nearly 90 per cent. of his autopsies. There are some resemblances between paresis and other forms of insanity, but with the patient's previous history and a careful analysis of the symptoms, a diagnosis can be arrived at when the proper management of our patients can be prepared for.

An ataxic gait with absence of the tendon reflexes and pupils which contract to light, but not to accommodation, will suggest tabes ; but if with these symptoms there is mental enfeeblement and hesitating speech, general paresis should be suspected and especially so when the lightning pains are absent. Again, disseminating sclerosis presents some similar symptoms, though the tremor is much coarser and there is more often nyatagmus ; there is not so marked ataxia and the mental symptoms appear much later. Paralysis agitans presents a plainly marked picture when one has studied these cases. I have diagnosticated several of these cases before the tremor began to show itself. The peculiar bent attitude, slow speech, the characteristic way of carrying the hand partially bent, bringing the thumb and finger together (this joint and others become fixed in many cases), the dull expressionless face, absence of pupillary alterations or of paroxysmal exacerbations and the constant tremor. No mental changes occur other than those accompanying old age.

Cases of melancholia, though similar to paresis in some respects, can after a little study and observation be diagnosticated. I have just sent to an institution a man who presented for six months a picture of stuporous melancholia with a tendency toward dementia. For only a month have I been at all suspicious of paresis. The depressed state has continued throughout the entire period, but the physical symptoms are developing and he has exhibited homicidal tendencies ; tendencies requiring moral restraint and separation from his family. In this case the cerebral reflexes were very slow. No grandiose ideas ; in fact, the depression and dulness were so marked that the physical signs were almost entirely masked. But recently the tremor, uncertain speech, skipping of syllables and letters, twitching of facial muscles and tremor of the tongue can be recognised, though only at intervals. There are no causes other than la grippe obtainable in this patient. The depression is intense in this class of cases, like an extreme type of melancholia, but the physical symptoms confirm the diagnosis.

Another form of paresis was presented in the following case :

Mr. B., 48 years of age, railroad engineer ; but of late a barkeeper. He gave a specific history, but was always robust. Symptoms—severe headache, nausea, tremor of the tongue, also of facial muscles, tremor of the hands, flushing and pallor of the face and contracted pupils. Following the prodromal or stage of alteration, he grew reckless, extravagant, suspicious and dishonest, finally homicidal. He had grandiose ideas and plans, relating to himself, his financial standing and physical development, requiring asylum care, where he has been for over two years. Convulsions threaten his life frequently. He has grown very stout and appears strong except when attempting any active movement, when tremor is very marked and exhaustion follows. He shows increased mental enfeeblement and will doubtless live only a few months.

Mr. S., aged 32 ; patient overworked and studied nights through his early life and always had heavy responsibilities and worries. He contracted syphilis some years ago, but was apparently well until the onset of what proved to be general paresis two years ago. When he first came under my care he complained of general weakness, forgetfulness, headache and general inability to do his work. Knowing something of his early history I prescribed the iodide and mercurial treatment with tonics. I also advised a vacation, which he took for one month, when he returned to the city greatly improved. In a few weeks the same story was repeated, with added distress that he failed to give satisfaction to his employer. At this time his pupils were irregular, one much smaller than the other and he began to be irritable and extremely feeble. Soon a convulsion occurred which completely unbalanced his reason ; he became unmanageable and was removed to an asylum. Typical symptoms developed, but none of the grandiose ideas. He passed from bad to worse and died recently in convulsions, only two years from the earliest noticed manifestations.

Time forbids the presentation of the histories of other cases under observation. Two of these given cases present syphilitic histories; in two there were no histories of specific disease, intemperance or traumatism; and as stated I find the minds of men divided regarding the etiology of this disease. All are convinced, however, of the advisability of an early diagnosis and close observations when the earliest symptoms become manifest, that by rest, electricity, tonic and specific treatment the progress may in some cases be delayed; also that bodily injury may be avoided and financial losses averted by checking extravagant investments and foolish schemes before it is recognised that our patient is mad.

505 Warren Street.

